# Diet Lists and Sick-Room Dietary

**Published:** 1901-01

**Authors:** 


DIET LISTS AND SICK-ROOM DIETARY. By JEROME B. THOMAS, M. D., Visiting Physician to the Home for Friendless Women and Children and to the Newsboys' Home; Assistant Bacteriologist, Brooklyn Health Department. Philadelphia: W. B. Saunders & Co. 1900. [Price, cloth, $1.50.]

The work contains 160 detachable (perforated) diet lists for albuminuria, anemia and debility, constipation, diabetes, diarrhea, dyspepsia, fevers, gout or uric-acid diathesis, obesity and tuberculosis. Also forty detachable sheets of sick-room dietary, containing full instructions for preparation of easily digested foods necessary for invalids. Each list is numbered only, an index key being reserved for the physician's private use.

The doctor will find the book a convenient labor-saving device.

